# Changes in antibiotic residues and the gut microbiota during ciprofloxacin administration throughout Silkie chicken development

**DOI:** 10.1016/j.psj.2022.102267

**Published:** 2022-10-23

**Authors:** Yushan Yuan, Peng Chen, Ying Li, Jiaheng Cheng, Xia Yan, Chenglong Luo, Dingming Shu, Hao Qu, Jian Ji

**Affiliations:** State Key Laboratory of Livestock and Poultry Breeding, Guangdong Key Laboratory of Animal Breeding and Nutrition, Institute of Animal Science, Guangdong Academy of Agricultural Sciences, Guangzhou, China

**Keywords:** ciprofloxacin, pharmacokinetics, gut microbiota, metabolism, Silkie chicken

## Abstract

The use of antibiotics leads to antibiotic residues in livestock and poultry products, adversely affecting human health. Ciprofloxacin (**CFX**) is a broad-spectrum antibiotic shared between animals and humans that is useful in treatments besides infections. However, changes in the gut microbiota caused by CFX and the possible link with the elimination of CFX residues have not been investigated. Herein, we used the Silkie chicken model to study the changes in the gut microbiota during the entire CFX-metabolic repertoire. We detected CFX residues in different tissues and showed that the elimination time of CFX from different tissues was dissimilar (liver > kidney > chest muscle > skin). Analysis of liver and kidney injury biomarkers and plasma antioxidant indices indicated slight hepatotoxicity and nephrotoxicity in the Silkie chickens. Importantly, the changes in the gut microbial community predominantly occurred early in the metabolic process. Correlation analysis revealed that the particular bacterial microbiota were associated with the pharmacokinetics of CFX in different Silkie chicken tissues (e.g., aerobic bacteria, including *Escherichia* and *Coprococcus*, and anaerobic bacteria, including *Fusobacterium, Ruminococcus, Bifidobacterium*, and *Eubacterium*). Collectively, certain microbiota may boost antibiotic metabolism and participate in restoring the microbial consortia after CFX is metabolized. Therefore, regulating the core intestinal microbiota may reduce foodborne antibiotics and accelerate the development of drug resistance.

## INTRODUCTION

The wide use of antibiotics in humans and livestock results in public health and safety risks, such as an increase of drug residues in animal-derived foods and antibiotic resistance (Kempf et al., 2015; Chen et al., 2019). Ciprofloxacin (**CFX**) is a representative fluoroquinolone antibiotic that is effective against several bacterial infections (WHO, 2020). Oral CFX is rapidly absorbed from the gastrointestinal tract and is mainly excreted via the kidneys (Cooper et al., 2007; Signore and Glaudemans et al., 2011; Schlender et al., 2018).

The gut microbiota has a symbiotic relationship with the host, which is a closely correlated/’[dynamic equilibrium (Groussin et al., 2020). The gut microbiota plays a critical role in human health by affecting various life activities, including defense against pathogens, development of the immune system, and regulation of drug disposition and pharmacokinetics (Shreiner et al., 2015; Kho and Lal, 2018; Schwartz et al., 2020; Tsunoda et al., 2021). Perturbations in the gut microbiota due to antibiotics result in many health problems, such as increased vulnerability to pathogenic bacteria and changes in metabolic pathways (Kuno et al., 2016; Binyamin et al., 2020). More than 50 drugs have been reported to participate in bidirectional interactions between the intestinal microbiota and drug metabolism (Altay et al., 2019).

CFX can induce adverse effects in the host, including destroying the integrity of the gut tract, reducing gut microbiota β-diversity, and disturbing the associations between key bacteria and metabolites (Dethlefsen and Relman, 2011; Zhu et al., 2020). In mice, CFX affects liver metabolism by reducing the lithocholic acid-producing intestinal microbiota (Toda et al., 2009). Previous studies have shown that several species of microbes are directly or indirectly involved in drug metabolism (Wilson and Nicholson, 2017; Zimmermann et al., 2019a; Javdan et al., 2020).

However, few studies have compared CFX metabolism with the composition of the intestinal microbiota. Here, we used the Silkie chicken model to show variations in the CFX concentrations in 4 tissues and the cecal microbiota composition after CFX treatment. We further found some core gut microbiota that may be involved in CFX metabolism in Silkie chickens. Taken together, the gut microbiota could serve as a potential manipulator of CFX metabolism in Silkie chickens to enhance the safety of meat and meat-based products.

## MATERIALS AND METHODS

### Ciprofloxacin

A soluble powder was obtained from Wens Dahuanong Biotechnology Co., Ltd. (Guangdong, China).

### Silkie Chicken and Experimental Design

The Silkie chicken are named for its atypically fluffy plumage that feels like silk or satin and originally bred in China. This study was conducted according to the Institutional Guidelines of Guangdong Province on the Review of Welfare and Ethics of Laboratory Animals and was approved by the Guangdong Province Administration Office of Laboratory Animals.

Three Silkie chickens were sacrificed 1 d before administering CFX (baseline, BL). A total of 448 female healthy Silkie chickens (30 d old) were randomly divided into the CFX-treated and control (CFX-free) groups consisting of 224 animals each. Silkie chickens in the CFX-treated group were administered 0.25 g/L CFX ad libitum supplemented in the water for 5 d. No Silkie chickens received additional antibiotics or probiotics before or during the study period. After the CFX treatment, 10 CFX-treated chickens and 6 control chickens were randomly selected and executed by bloodletting of the jugular vein at 14-time points (at h 4, and on d 1, 3, 5, 7, 9, 12, 20, 30, 40, 50, 60, 75, and 120). Individual blood samples were collected from the jugular vein into sodium heparinized tubes, and plasma samples were separated by centrifugation at 4,000 rpm for 8 min at 4°C and stored at −80°C for analysis. The chest skin (including subcutaneous fat with the feathers removed), entire left chest muscle, liver, and kidneys were separated and taken with a sterile and CFX-free scalpel or scissors and stored at −80°C until further processing for the pharmacokinetic analysis. The ceca of all Silkie chickens were opened with scissors, and the cecal digesta was removed with a sterilized 1 mL pipette. The cecal digesta was collected in plastic RNAse and DNAse-free tubes, snap-frozen in liquid nitrogen, and stored at −80°C until further analyses.

### Determination of CFX Concentrations

High-performance liquid chromatography-mass spectrometry (HPLC-MS) was used to quantify the CFX concentrations in four tissues (chest muscle, skin, kidney, and liver) from the treated Silkie chickens. Based on the internal standard method, 1.0 g of minced and mixed tissue was weighed, and 20 μL of 200 ng/μL D8-CFX (Dr. Ehrenstorfer GmbH, Mannheim, Germany) was added and equilibrated at room temperature for 10 min. The extraction procedure included adding 5 mL of acetonitrile containing 1% formic acid, and vortexing, followed by centrifugation at 8,000 rpm for 5 min. The extract was transferred to a 50 mL centrifuge tube and blown dry under a stream of nitrogen gas. The residue was dissolved in 1 mL of 1% formic acid/acetonitrile (9:1, v/v) and 3 mL of 100% hexane, mixed, and allowed to separate. The sublayer was extracted, centrifuged at 10,000 rpm for 2 min, and filtered through a 0.22-μm nylon filter for the HPLC-MS analysis.

The HPLC-MS system was controlled using a Shimadzu LC-MS 8050 system (Shimadzu, Tokyo, Japan) equipped with a CBM 20A controller, a pump (LC 30AD), a degasser (DGU 20A), a detector (SPD M30A), an autosampler (SIL 30AC), a column oven (CTO 30A), and a mass spectrometer (LCMS 8050) to analyze the CFX concentrations. Chromatographic separation was achieved with a Kinetex C18 column (100 × 2.1 mm i.d., 2.6 μm; Phenomenex, Torrance, CA), whose temperature was held at 40°C. An external calibration curve was created by injecting 5 μL of 10, 20, 50, 100, 200, 500, or 1,000 ng CFX standards. The mobile phase consisted of 0.1% formic acid and methanol (8:2) at a flow rate of 0.3 mL/min. A full scan in positive ion mode was used to record the mass spectra under the infusion mode, and the target compounds were quantified at m/z 332.1 and m/z 340.1 for CFX and D8-CFX, respectively. The LabSolutions software program was used to control the instrument and analyze the data.

### Biochemical Analysis

The plasma (350–400 µL) samples were centrifuged at 4,000 rpm for 10 min. The supernatant was collected and stored at 4°C until the assay. The activities of alanine aminotransferase (**ALT**), albumin (**ALB**), uric acid (**UA**), and creatinine (**CREA**) were determined spectrophotometrically in plasma using the Chemray 800 Biochemistry autoanalyzer and kits (Rayto Life and Analytical Sciences Co., Ltd., Shenzhen, China). Levels of total bile acids (**TBA**s) were examined with the Chemray 800 Biochemistry autoanalyzer using a kit from Changchun Huili Biotech (Changchun, Jilin, China) according to the manufacturer's instructions. Glutathione peroxidase (**GSH-Px**) was analyzed with an assay kit (Nanjing JianCheng Bioengineering Institute, Nanjing, China) following the instruction manual.

### Microbial Genomic DNA Extraction

Bacterial DNA for the 16S rDNA polymerase chain reaction (**PCR**) was extracted from the cecal digesta using the Mag Pure Stool DNA KF kit B (Magen, Shanghai, China) following the manufacturer's protocol. The Qubit fluorometer and the Qubit dsDNA BR Assay kit (Invitrogen, Carlsbad, CA) were used to quantify the DNA, and DNA quality was checked by 1% agarose gel electrophoresis. The DNA extracted from all samples was stored at −20°C until sequencing.

### Microbial 16S rDNA Gene Sequencing

Binding sites for sequencing the BGI-seq500 primers were added at the 5′ end of the common primers 515F (5′- GTGCCAGCMGCCGCGGTAA-3′) and 806R (5′-GGACTACHVGGGTWTCTAAT-3′). The following PCR conditions were used: 5 min of denaturation at 94°C, 10 cycles of 30 s at 94°C, 30 s of annealing at 60°C, 30 s of elongation at 72°C, and a final extension at 72°C for 10 min. After two amplifications, the PCR products were extracted from a 2% agarose gel, purified using the QIAquick Gel Extraction kit (Qiagen, Hilden, Germany), and quantified using the Qubit dsDNA HS Assay Kit (ThermoFisher, Waltham, MA) according to the manufacturer's protocol. The purified amplicons were pooled in equimolar concentrations and sequenced paired-end read on the MGISEQ-2000 platform (BGI, Qingdao, China).

### Sequencing Data Analysis

The raw reads were processed to eliminate adapter sequences and low-quality regions. Paired-end sequence reads were merged using FLASH (Fast Length Adjustment of Short reads, v1.2.11) (Magoč and Salzberg, 2011). The sequences were clustered into operational taxonomic units (**OTU**s) at a 97% identity threshold using USEARCH (v7.0.1090) (Edgar R, 2013). Taxonomic assignment of each OTU was made using the Ribosomal Database Project (**RDP**) classifier and scripts in QIIME (v1.8.0) software (Cole et al., 2014). The Venn diagram and the Upset plot were drawn using TBtools (version 1.068). α-Diversity metrics (observed OTU richness, Chao1 richness, and the Shannon and Simpson diversity indices) were calculated from the rarefied libraries using Mothur (v1.31.2), and the rarefaction curves were drawn using R (v3.4.1) software. The β-diversity analysis (unweighted UniFrac and weighted UniFrac) was performed using QIIME (v1.80) software. Principal component analysis (**PCA**) was conducted using the PCA in R 3.4.1 with the “vegan” package (The R Foundation for Statistical Computing, Vienna, Austria).

### Statistical Analysis

Statistical analyses were performed using Prism software v. 8.0 (GraphPad Software Inc., La Jolla, CA). Blood biochemical indicators between the CFX treatment and controls were assessed using Student's *t*-tests. *P*-values < 0.05 were considered significant. The α-diversity, β-diversity, and taxa abundances at the phylum and generic levels between the two groups were made with the Wilcoxon test, and visualized with box-and-whisker plots. Differences in microbial composition between the groups were assessed by permutational multivariate analysis of variance (PERMANOVA, ‘adonis’ function, R vegan package). *P*-values were corrected for multiple samplings using the Benjamini–Hochberg false discovery rate procedure with the p.adjust function in R.

## RESULTS

### CFX Residues in the Tissue Samples

**Figure 1:**
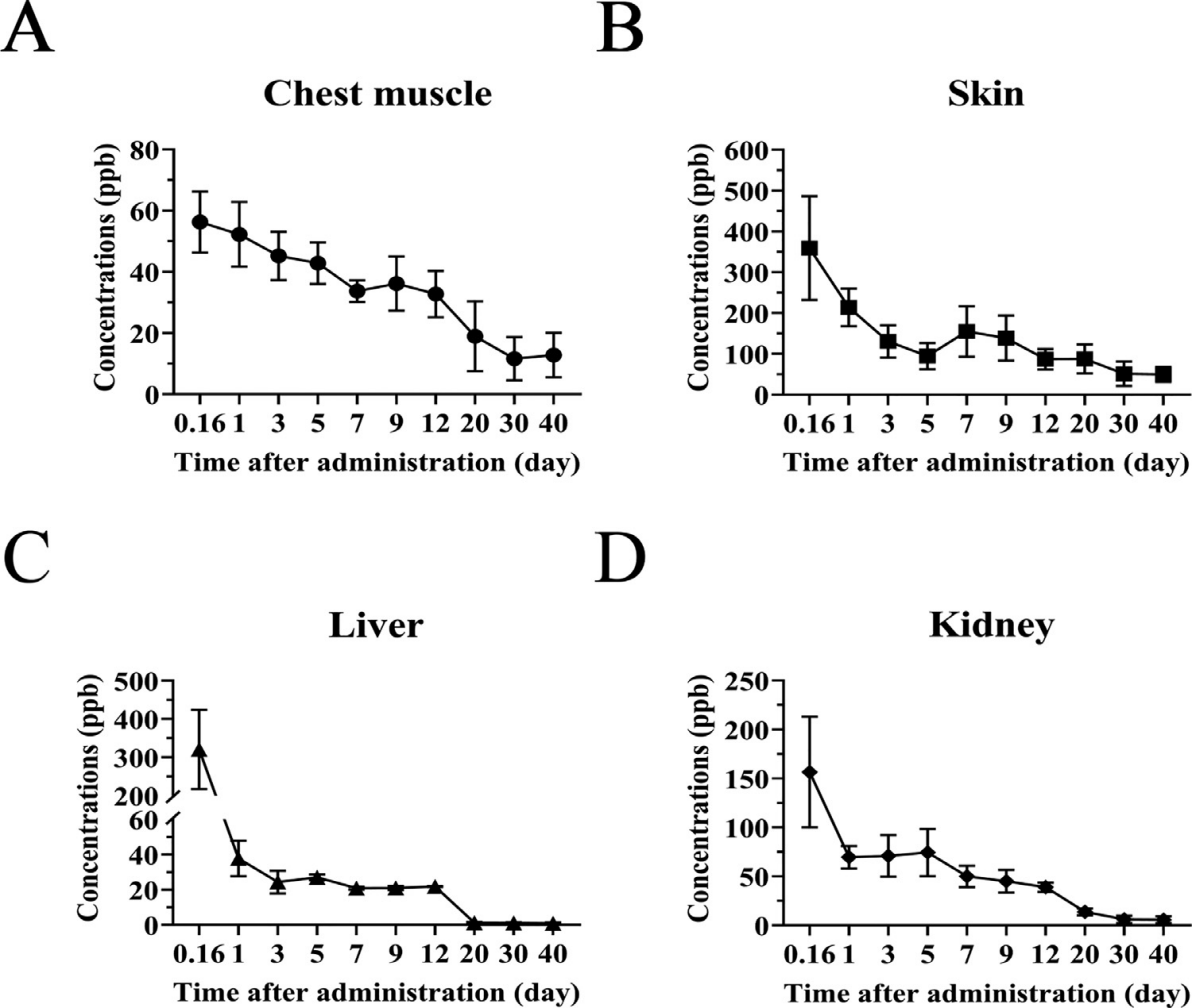
The pharmacokinetics of CFX after administration to Silkie chickens. (A) The concentrations of CFX in chest muscle. (B) Skin. (C) Liver. (D) Kidney. N ≥ 8 for each group, and data were shown as mean ± SEM.

CFX residue levels in the four tissues (chest muscle, skin, liver, and kidney) were measured by HPLC-MS (Figure 1A–D). CFX residue levels were the highest on d 0.16 after CFX was administered and decreased over time. Among the 4 tissues, CFX residue levels were highest in the skin (359.18 ± 126.83 µg/kg), followed by the liver (320.78 ± 103.59 µg/kg), kidneys (156.60 ± 56.48 µg/kg), and chest muscle (56.27 ± 9.97 µg/kg). The CFX concentration in the liver decreased rapidly over time and was undetectable by HPLC-MS on d 30 after the CFX treatment. However, on d 30, CFX was highest in skin tissues and exceeded the maximum residue limit (**MRL**). Forty days after the CFX treatment, CFX residue levels in all tissues were very low (below the MRL) and insignificant. Notably, CFX residue levels were highest in the skin at the last sampling time point (49.79 ± 18.61 µg/kg), followed by the chest muscle (12.86 ± 7.27 µg/kg), the kidney (5.66 ± 3.57 µg/kg), and the liver (0.86 ± 0.49 µg/kg, respectively). Our data suggest that the CFX residual time was long in the Silkie chicken skin.

### The Effect of CFX on Blood Biochemical Parameters

**Figure 2:**
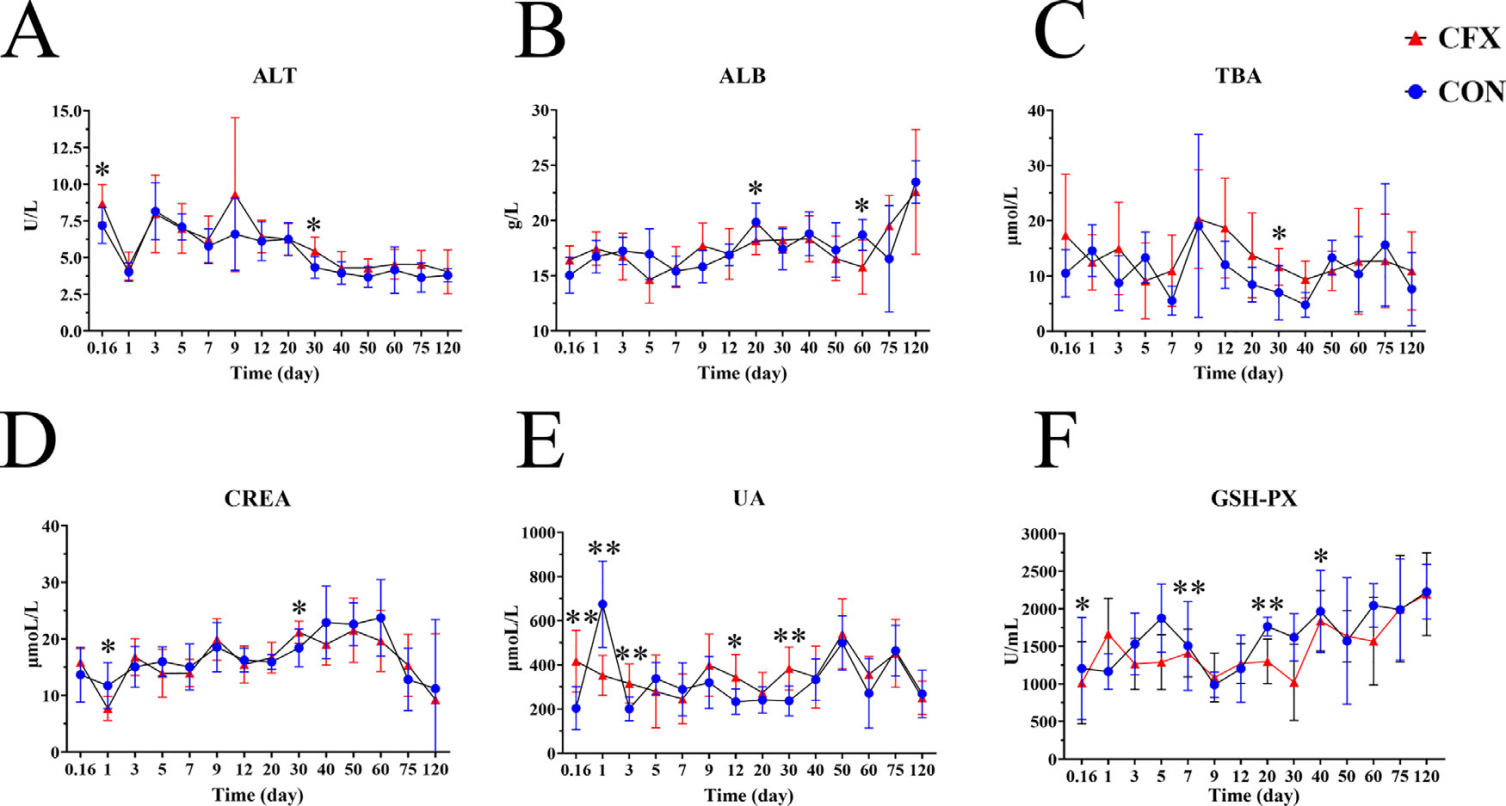
Silkie chicken biochemistry parameters of the two groups after stopping CFX. (A) Change in alanine aminotransferase in Silkie chicken serum (ALT). (B) Serum albumin (ALB). (C) Total bile acids (TBA). (D) Creatinine (CREA). (E) Blood uric acid (UA). (F) Glutathione peroxidase (GSH-Px). * *P <* 0.05, ** *P <* 0.01; unpaired *t*-test. Ctr, controls (n ≥ 5); CFX, CFX treated Silkie chickens (n ≥ 8).

We further analyzed the blood biochemical indices of the Silkie chickens after the CFX treatment. Here, we detected hepatic (ALT, ALB, and TBA) and renal function indicators (UA and CREA) as well as serum antioxidant capacity (GSH-Px). As shown in Figure 2A, the CFX treatment resulted in significantly higher serum ALT levels on d 0.16 and 30 than those in the control group (*P <* 0.05). The CFX treatment reduced serum ALB levels (on d 20 and 60, *P <* 0.05) and increased TBA (on d 30, *P <* 0.05) (Figure 2B, C). Interestingly, GSH-Px activity decreased significantly in the CFX treatment group on d 0.16, 7, 20, and 40 (*P <* 0.05) while on day 1 it tended to increase (*P* > 0.05) compared to the control group (Figure 2F). Serum CREA levels in the CFX treatment group decreased significantly on d 1 compared to the control group, while they increased significantly on d 30 (*P <* 0.05) (Figure 2D). UA in the CFX treatment group was significantly higher than that in the control group on d 0.16, 3, 12, and 30, but lower on d 1 (*P <* 0.05) (Figure 2E). Collectively, these data suggested that CFX induced the increases in of ALT, TBA, UA, and CREA may cause by the effect of CFX on liver and kidneys.

### The Effect of CFX on Overall Cecal Microbiota Structure

**Figure 3:**
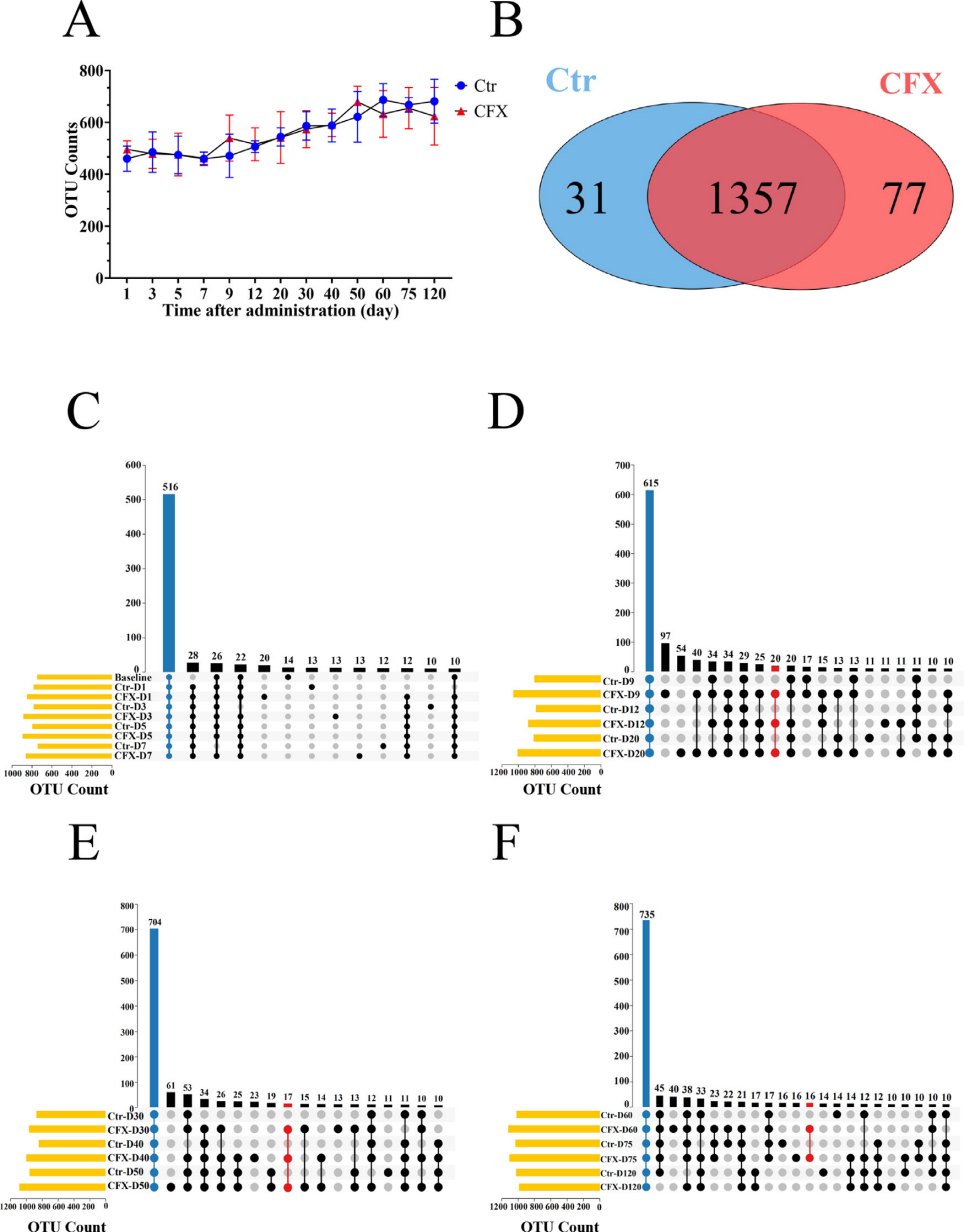
Changes in the Silkie chicken gut microbiota at the OTU level. (A) OTU counts of CFX-treated and the control (Ctr) at different times. (B) Differences in OTUs between the Ctr and CFX groups throughout the trial period were analyzed using Venn diagrams. (C–F) The UpSet Plot of the distribution of OTUs at different withdrawal times in CFX-treated Silkie chickens. D: day. Ctr, controls (n ≥ 5); CFX, CFX treated Silkie chickens (n ≥ 8).

16S rDNA gene sequencing was performed to characterize the changes in the gut microbiota after the CFX treatment at different time points. The number of operational taxonomic units (**OTU**s) was not significantly different between the CFX treatments and control groups on d 1 and 120 (Figure 3A). As shown in the Venn diagram (Figure 3B), the CFX treatment group had 77 unique OTUs, the control group had 31 unique OTUs, and 1,357 OTU were shared between the two groups. The Upset Plot shows that the number of intersections between the CFX treatment and control groups increased with time, while the number of CFX treatment group unique OTUs gradually decreased from d 9 to d 120 (Figure 3C–F). Interestingly, the number of unique OTUs in the CFX treatment group increased initially, reached a peak on d 9 (157 OTU), and then decreased gradually from d12 to d 120. On d 120, there were only 10 unique OTUs in the CFX treatment group. Our analysis revealed that CFX induced changes in the overall structure of the cecal microbiota.

### Dynamics of Cecal Microbiota β-diversity After the CFX Treatment

**Figure 4:**
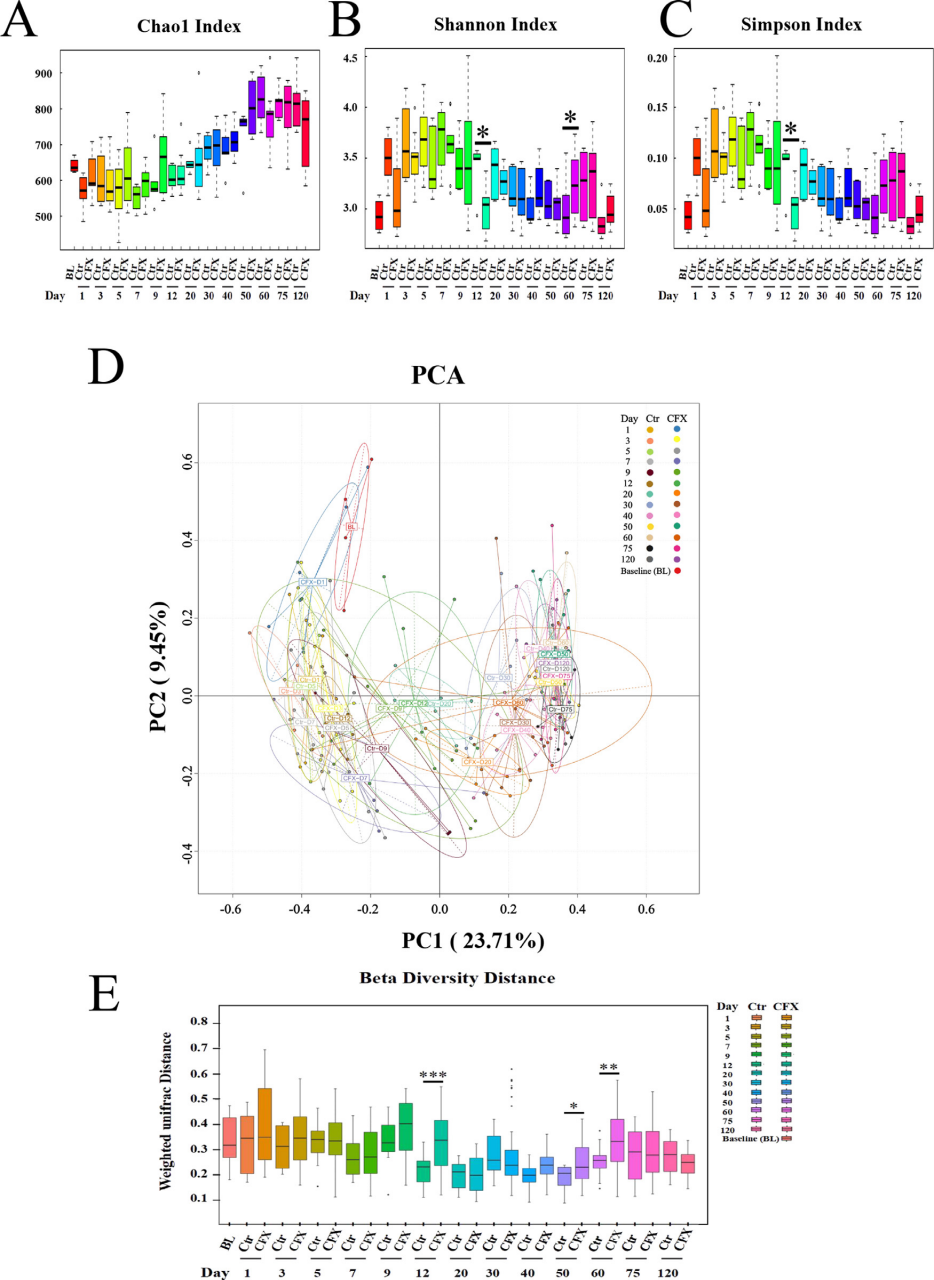
Effects of the CFX treatment on the diversity of the gut microbiota. (A) Differences in bacterial richness between the control and CFX-treated Silkie chickens at different times were analyzed with the Chao1 index. (B) Differences in bacterial α-diversity between the two groups were analyzed with the Shannon index. (C) Differences in β-diversity between the Ctr and CFX groups at different times were analyzed with principal component analysis. (D) The weighted unifrac distance between the Ctr and CFX groups. * *P <* 0.05, ** *P <* 0.01, *** *P <* 0.001; Wilcoxon rank-sum test. Ctr, controls (n ≥ 4); CFX, CFX treated Silkie chickens (n ≥ 6).

α-Diversity and β-diversity metrics were analyzed to explore the effect of CFX on microbial β-diversity in the Silkie chicken cecum (Figure 4A–E). The richness index (Chao1) for birds did not differ between the two groups (Figure 4A). However, there were significant differences in the Shannon index between the groups on d 12 and 60 and a significant difference only in the Simpson index on d 12 (*P <* 0.05) (Figure 4B, C). The β-diversity result was measured by PCA, which explained 23.71% (PC1) and 9.45% (PC2) of the total variance (Figure 4D). The CFX and control groups gradually separated from d 1 to 9, clearly separated from the control group on d 12, and then the gap gradually narrowed from 20 to 120. In this experiment, the gut microbiota ecosystem developed towards a steady state in the 3 d after the antibiotic treatment and no clear clusters were present for either group on day 120. Although CFX was separated from the controls site at some time points, it partially overlapped with the controls. Based on the unweighted UniFrac distances, CFX significantly altered the microbial community structure on d 12, 50, and 60 (*P <* 0.001, *P <* 0.05, *P <* 0.01, respectively) (Figure 4E). These temporal changes indicated that CFX suppressed multiple taxa early and changed the overall diversity of the cecal microbiota.

**Figure 5:**
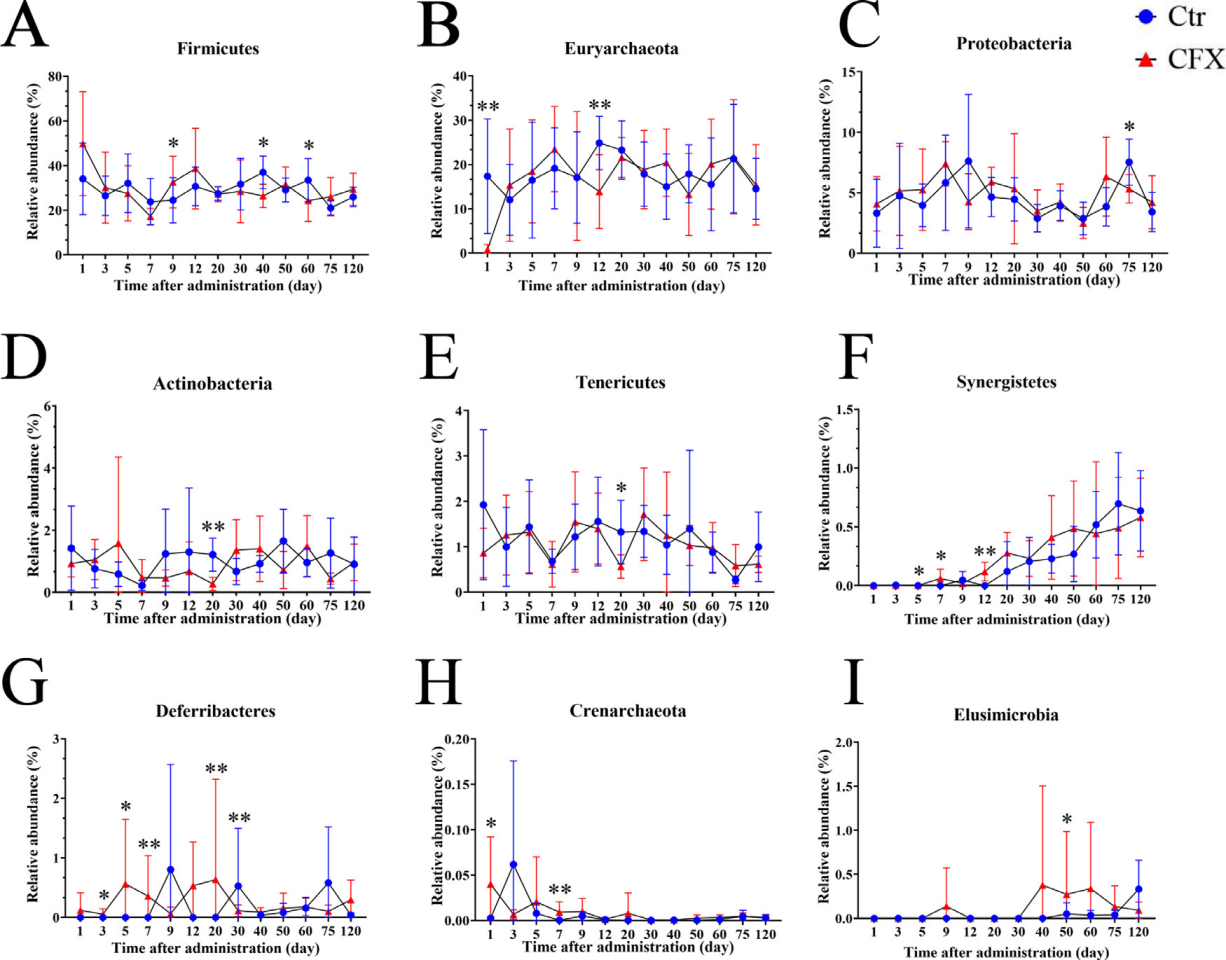
Relative abundances of the gut microbiota at the phylum level showing significant changes in response to the CFX treatment during the early period after withdrawal. (A) Differences in the relative abundance of *Firmicutes* between the control and the CFX treated group. (B) The relative abundance of *Euryarchaeota*. (C) The relative abundance of *Proteobacteria*. (D) The relative abundance of *Actinobacteria*. (E) The relative abundance of *Tenericutes*. (F) The relative abundance of *Synergistetes*. (G) The relative abundance of *Deferribacteres*. (H) The relative abundance of *Crenarchaeota*. (I) The relative abundance of *Elusimicrobia*. * *P <* 0.05, ** *P <* 0.01; Wilcoxon rank-sum test. Ctr, controls (n ≥ 4); CFX, CFX treated Silkie chickens (n ≥ 6).

### Microbiota Compositional Changes During the CFX Withdrawal Time at the Phylum Level

We assessed the relative abundance of microbial species in the two groups at the phylum level. The relative abundances of the microbial species changed over time and differed by age. Twenty-five phyla were detected in the bacterial communities of the Silkie chickens, which were dominated by *Bacteroidetes* (43% on average), *Firmicutes* (29%), *Euryarchaeota* (18%), and *Proteobacteria* (5%) (Figure S1). The abundances of *Crenarchaeota* (on d 1 and 7), *Synergistetes* (on d 5, 7, and 12), and *Elusimicrobia* (on d 50) increased significantly in the CFX treatment group, compared to the control group. In contrast, the abundances of *Euryarchaeota* (d 1 and 12), *Actinobacteria* (d 20), *Tenericutes* (d 20), and *Proteobacteria* (d 75) decreased significantly (*P <* 0.05) as indicated in Figure 5A to I. Interestingly, the abundance of *Firmicutes* increased significantly on d 9 and then decreased on d 40 and 60 in the CFX treatment group, compared to the control group (*P <* 0.05). Moreover, the abundance of *Deferribacteres* increased first (on d 3, 5, 7, and 20) and then decreased on day 30, compared to the control group (*P <* 0.05). These results indicated that the effect of CFX on the composition of the gut microbiota was rapid and profound after the treatment.

### The Effect of CFX Treatment on Microbiota Composition at the Genus Level

**Figure 6:**
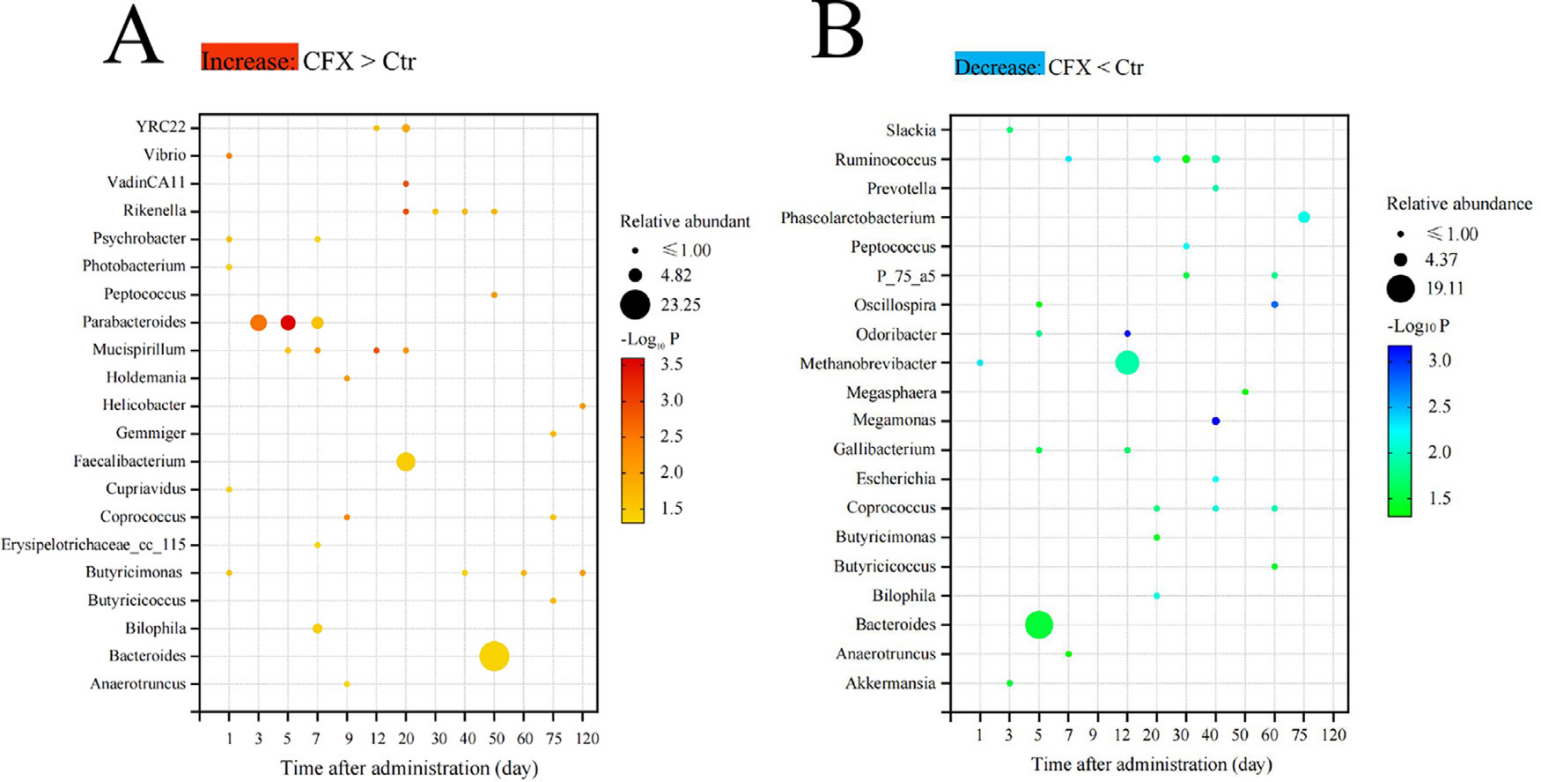
The relative abundances of the gut microbiota changed significantly in response to the CFX treatment. (A) The relative abundance of the gut microbiota increased in the CFX-treated group, compared to the control group. (B) The relative abundance of the gut microbiota decreased in the CFX-treated group, compared to the control group. Wilcoxon rank-sum test. Ctr, controls (n ≥ 4); CFX, CFX treated Silkie chickens (n ≥ 6).

The dominant genera in each identified cluster were *Bacteroides* (19.5% on average), *Methanobrevibacter* (17.8%), *Faecalibacterium* (4.5%), *Phascolarctobacterium* (3.6%), *Desulfovibrio* (1.9%), *Megamonas* (1.5%), *Oscillospira* (1.4%), and *Lactobacillus* (1.21%) (Figure S2). Fifty-five genera changed significantly after the CFX treatment (34 genera with relative abundance > 0.05%, *P <* 0.05), and many of them were presented during the early stage (12 d after CFX stopped). Focusing on the early stage, compared to the control group, many common taxa, such as *Bilophila, Butyricimonas, Erysipelotrichaceae_cc_115, Coprococcus, Cupriavidus, Mucispirillum*, and *Parabacteroides*, as well as the pathogens *Holdemania, Photobacterium, Psychrobacter*, and *Vibrio* increased in abundance in the CFX group (Figure 6A) In contrast, less common microbiota, such as *Akkermansia, Bacteroides, Methanobrevibacter, Oscillospira, Odoribacter, Ruminococcus*, and *Slackia* decreased in abundance (*P <* 0.05). Species were only different on d 120 (*P <* 0.05) (Figure 6B). These results demonstrated that the differences in gut microbiota between the CFX-treated and control groups mainly appeared during the early stage.

### Correlation Between the Gut Microbiota and CFX Residue Levels in Different Tissues

**Figure 7:**
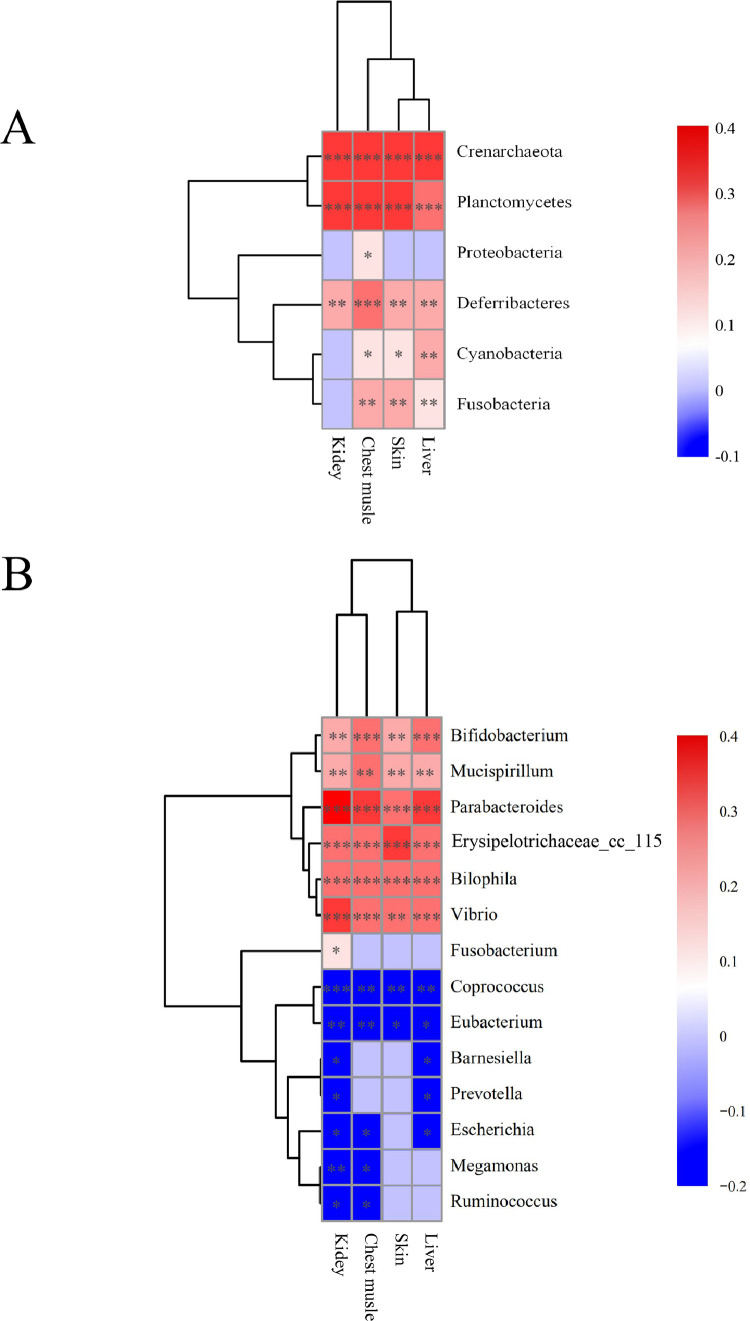
The gut microbiota and residual CFX in different tissues were significantly correlated. (A) Pearson's correlation of the gut microbiota and CFX residues at the phylum level. (B) Pearson's correlation of the gut microbiota and CFX residues at the genus level. * *P <* 0.05, ** *P <* 0.01, *** *P <* 0.001; Pearson's test. Pearson's correlation coefficients (R) are shown as values and the corresponding *P*-value of the Pearson's linear correlation (P) is indicated by *. Ctr, controls (n ≥ 4); CFX, CFX treated Silkie chickens (n ≥ 6).

Pearson's correlation analysis and Student's *t*-test revealed a strong correlation between CFX residues in the Silkie chickens and the composition of the cecal microbiota. The relative abundances of several bacterial phyla, including *Crenarchaeota, Cyanobacteria, Deferribacteres, Fusobacteria, Planctomycetes*, and *Proteobacteria* were positively correlated with CFX residues levels in the different tissues (*P <* 0.05). However, no negative correlations were detected (Figure 7A). The relative abundances of several bacterial genera, including *Bifidobacterium, Bilophila, Mucispirillum, Parabacteroides, Vibrio, Erysipelotrichaceae_cc_115*, and *Ruminococcus*, were positively correlated with CFX residue levels in the 4 tissues (*P <* 0.05), but *Fusobacterium* was only correlated in the kidney. In contrast, the relative abundances of *Coprococcus, Eubacterium, Barnesiella, Prevotella, Escherichia, Megamonas*, and *Ruminococcus* were negatively correlated with CFX residue levels in the kidney (*P <* 0.05). Notably, CFX residue levels in the kidney were negatively correlated with *Barnesiella, Escherichia, Megamonas, Prevotella*, and *Ruminococcus* (r = −0.176, −0.180, −0.211, −0.172, and −0.202, *P <* 0.05 respectively) in the Silkie chickens (Figure 7B). These close correlations suggested that the gut microbiota may participate in CFX metabolism in Silkie chickens, particularly in the kidney.

## DISCUSSION

Antibiotics are widely used to treat livestock diseases. However, the metabolism of antibiotics is incomplete, leading to residues in animal-derived foods, disrupting the intestinal flora, and endangering the health and safety of consumers (Bacanlı and Başaran, 2019). In the present study, CFX mainly accumulated in the liver, skin, and kidney of Silkie chickens and profoundly affected the intestinal flora. To clarify the effect of the CFX residues, we further investigated the changes in blood biochemical parameters over time and screened the gut microbiota associated with CFX metabolism. The CFX concentration in the skin of one individual that was detected 30 d after the CFX treatment was more than the MRL (100 μg/kg), suggesting that CFX was eliminated slowly, particularly from the skin. Low CFX residue concentrations were detected in the liver, chest muscle, kidney, and skin from some individuals by HPLC-MS 40 d after administration. Unlike in broiler chickens, CFX and its metabolites were rapidly cleared within 10 d of administration (Anadón et al., 2001).

CFX disrupted homeostasis in Silkie chickens to provide a window for the proliferation of pathogens, including altered cecal microbiota composition and increased susceptibility to infection. The 16S rDNA gene sequencing analysis showed that the relative abundance of phylum *Synergistetes* increased significantly in the CFX-treated group, compared to the controls. *Synergistetes* bacteria are anaerobic and significantly correlated with the antibiotic resistance gene (Ma et al., 2021). *Synergistetes* have been suggested to be opportunistic pathogens and have been implicated in several infectious diseases (Horz et al., 2006; Vartoukian et al., 2007; Marchandin et al., 2010). Furthermore, the inflammation-mediator *Mucispirillum*, among other pathogenic bacteria (Shenghua et al., 2020), tended to increase like *Synergistetes*. Our study showed that the CFX treatment caused dysbiosis, even after a short-term treatment, which had a profound and lasting effect on the distal gut microbiota, consistent with the findings of Dethlefsen L (Dethlefsen and Relman, 2011).

Administering CFX can lead to various adverse events, including abnormal liver function tests, drug-induced liver injury, and interstitial nephritis (Owens and Ambrose, 2005; Chalasani et al., 2008; Drug Safety and Availability – FDA Drug Safety Communication, 2016). The CFX-treated Silkie chickens had higher serum ALT and TBA levels than the control group, which may have been caused by the destruction of hepatocyte structural integrity (Jayakumar et al., 2020). The kidney indicators (CREA and UA) increased significantly in our study and have been previously reported in CFX-induced kidney injured patients (Moffett et al., 2003; Goli et al., 2017). GSH-Px activity decreased in the CFX group likely because the drug concentration was more than the GSH-Px capacity (Gao et al., 2013). Our results paralleled the finding that broad-spectrum antibiotics deplete the gut microbiome, impair intestinal barrier function, increase intestinal permeability, and damage the antiviral immune defense in the liver (Guo et al., 2021).

The gut microbiota contributes to the distribution, metabolism, and excretion of host drugs (Tsunoda et al., 2021). Here, *Fusobacteria* was positively correlated with the elimination of CFX. *Fusobacteria* produce the enzyme azoreductase and convert the immunosuppressant tacrolimus to less potent metabolites (Guo et al., 2019). *Parabacteroides* may be the most prominent driver of CFX disposition and metabolism, as it was correlated with the concentrations of CFX in all four tissues. *Parabacteroides* are known as inflammatory bacteria and are associated with diarrhea and the early stages of cirrhosis (Ren et al., 2019; Yue et al., 2019; Li et al., 2020). *Parabacteroides* produce β-glucuronidase, which is involved in drug efficacy and toxicity (Little et al., 2018). *Coprococcus* may play an essential role in CFX metabolism and excretion in Silkie chickens. A previous study showed that *Coprococcus* participates in drug metabolic activities in *ex vivo* co-cultures (Zimmermann et al., 2019b). However, further experiments are needed to provide evidence for this study, including transplanting fecal bacteria and editing the genes of a single bacterium.

Numerous studies have confirmed that residual antibiotics in food can harm humans, including but not limited to antimicrobial resistance and dysfunction of beneficial gut microbiota. We identified candidate microbiota members that promoted CFX metabolism in Silkie chickens by combining pharmacokinetics with high-throughput microbial 16S rDNA profiling. Therefore, CFX may have the potential to be targeted therapeutically as a residual antibiotic in animals.

## CONCLUSION

In summary, the results of this study indicate that CFX residues in different tissues are tightly associated with certain gut microbiota, which could serve as a potential manipulator of CFX metabolism in Silkie chickens to enhance the safety of meat and meat-based products.

## Appendix Supplementary materials

Image, image 1

Image, image 2

## Footnotes

Supplementary material associated with this article can be found, in the online version, at doi:10.1016/j.psj.2022.102267.
