# Supplementary figures and images for: Changes in antibiotic residues and the gut microbiota during ciprofloxacin administration throughout Silkie chicken development
Source: Poult Sci. 2022 Oct 23;102(1):102267. doi: 10.1016/j.psj.2022.102267 (PMC9709234; doi:10.1016/j.psj.2022.102267)

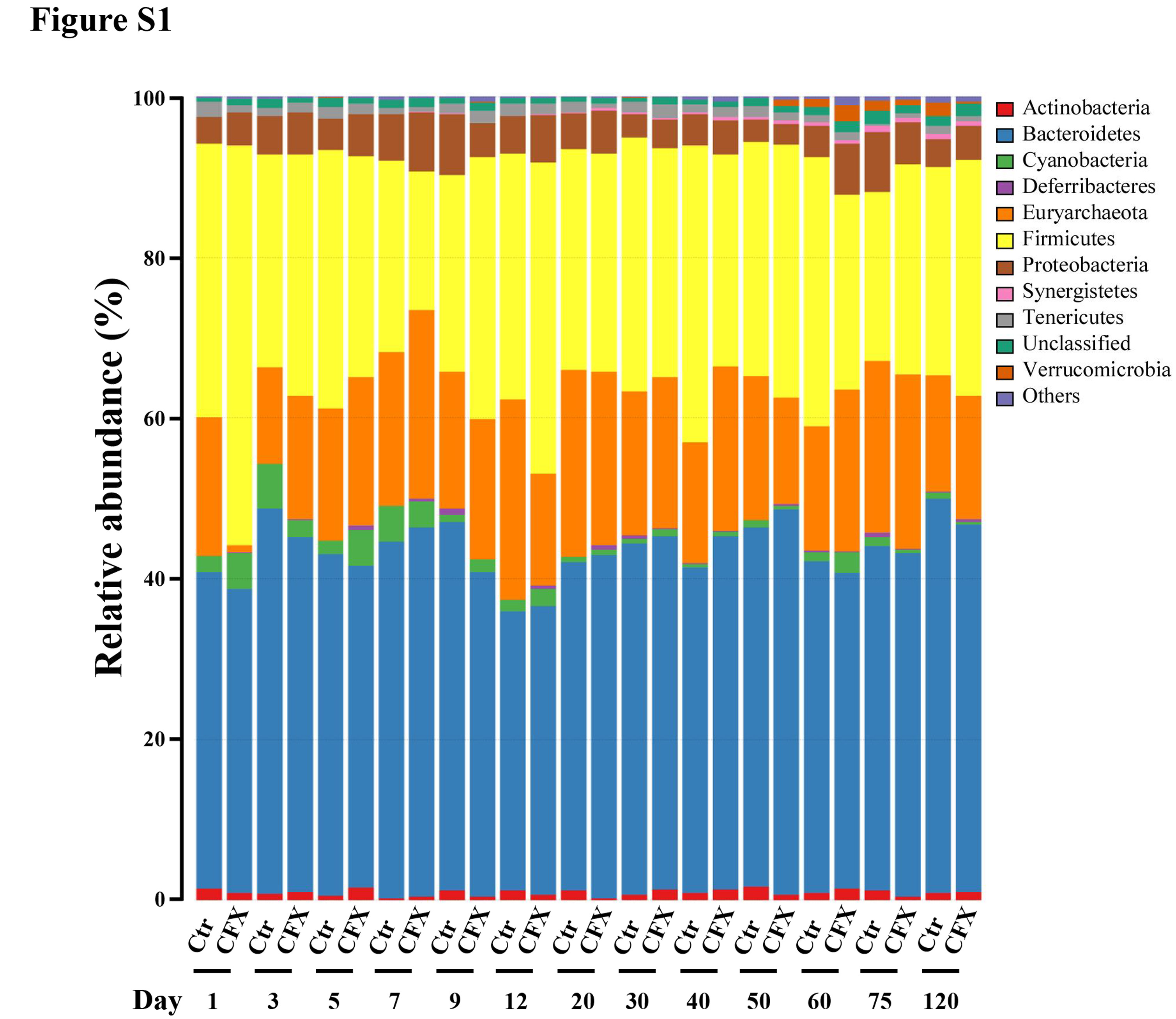

Supplement: Supplementary file 1 [file mmc1.jpg]

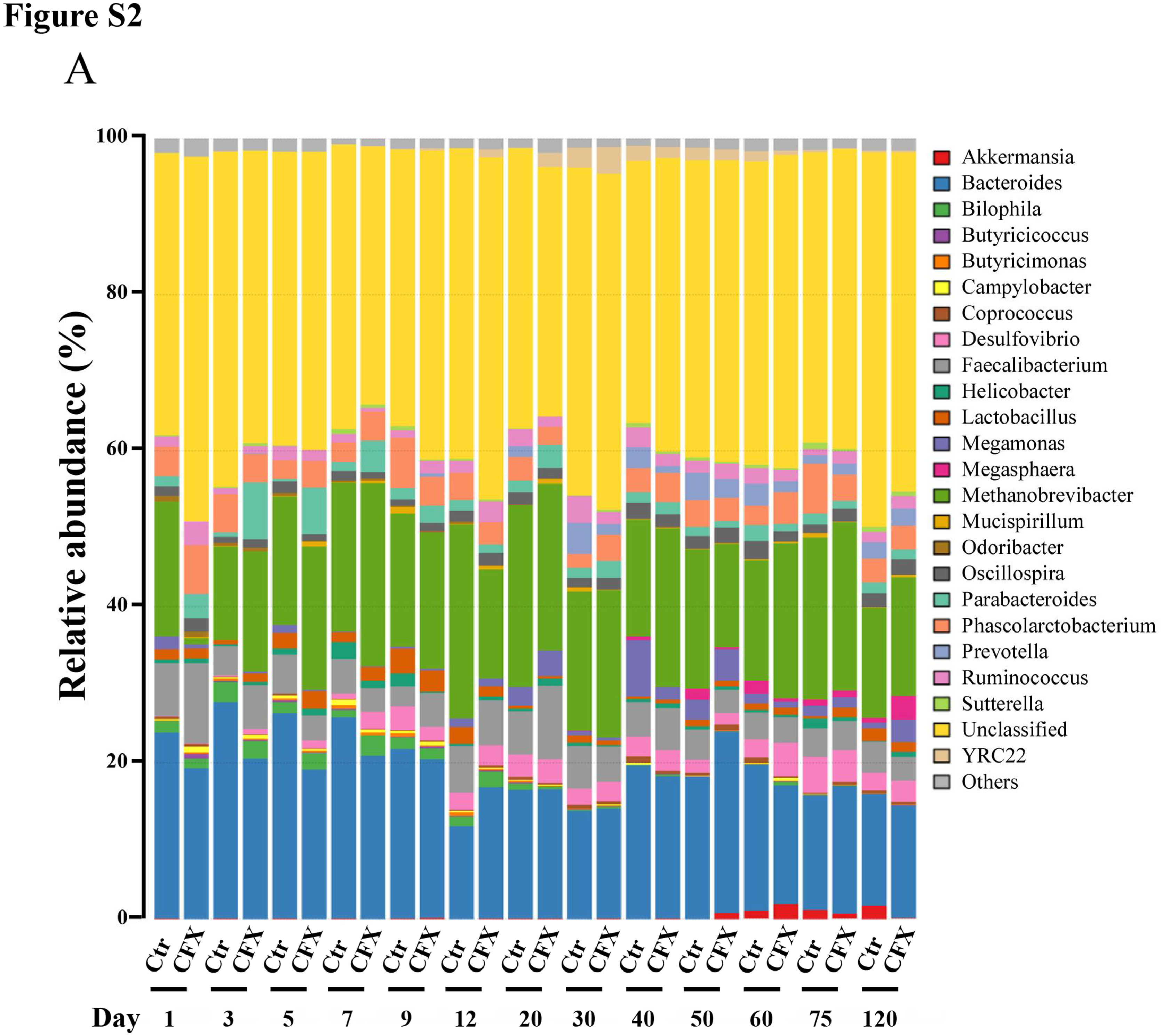

Supplement: Supplementary file 2 [file mmc2.jpg]
